# Analysis of particulate exposure during continuous drug infusion in critically ill adult patients: a preliminary proof-of-concept in vitro study

**DOI:** 10.1186/s40635-018-0205-2

**Published:** 2018-10-11

**Authors:** Malik Benlabed, Anthony Martin Mena, Romain Gaudy, Maxime Perez, Stéphanie Genay, Jean-Daniel Hecq, Pascal Odou, Gilles Lebuffe, Bertrand Décaudin

**Affiliations:** 1 Univ. Lille, EA 7365-GRITA-Groupe de Recherche sur les formes Injectables et les Technologies Associées, F-59000 Lille, France; 2 CHU Lille, Pharmacie, F-59000 Lille, France; 3 Department of Pharmacy, CHU UCL Namur, avenue Therasse, 1, 5530, Yvoir, Belgium; 4 Drug Stability Research Group, CHU UCL Namur, avenue Therasse, 1, 5530, Yvoir, Belgium; 5 CHU Lille, Pôle d’Anesthésie-Réanimation, F-59000 Lille, France

**Keywords:** Parenteral nutrition, Intravenous, Infusion pumps, Drug incompatibility, Critical care

## Abstract

**Background:**

In critically ill patients, drug incompatibilities frequently occur because of the number of drugs to be administered through a limited number of infusion lines. These are among the main causes of particulate contamination. However, little data is available to quantify particle exposure during simultaneous IV-drug infusion. The objective of this study was to evaluate the particulate matter potentially administered to critically ill patients.

**Methods:**

The particulate matter (between 1 μm and 30 mm) of infused therapies used in ICUs for patients suffering from either septic shock or acute respiratory distress syndrome was measured in vitro over 6 h using a dynamic image analysis device, so that both overall particulate contamination and particle sizes could be determined. Data is presented according to the recommendations of the European Pharmacopoeia (≥ 10 and 25 μm).

**Results:**

For the six experimental procedures (continuous infusion of norepinephrine, midazolam, sufentanil, heparin, 5% glucose, binary parenteral nutrition and discontinuous administrations of omeprazole, piperacillin/tazobactam and fluconazole), the overall number of particles over the 6-h infusion period was 8256 [5013; 15,044]. The collected values for the number of particles ≥ 10 and 25 μm were 281 [118; 526] and 19 [7; 96] respectively. Our results showed that discontinuous administrations of drugs led to disturbances in particulate contamination.

**Conclusions:**

This work indicates the amount of particulate matter potentially administered to critically ill adult patients. Particulate contamination appears lower than previous measurements performed during multidrug IV therapies in children.

## Background

Little data is available on the clinical implications of intravenous drug incompatibilities in critically ill patients [1]. Drug incompatibilities via particulate contamination may increase the risk of organ dysfunction [1]. Randomised controlled trials comparing infusion with or without filter have yielded mixed results. One study conducted in a paediatric intensive care unit (ICU) reported a reduction in the incidence of severe complications in the filter group [2]. Two studies performed on neonatal ICU patients were in disagreement over the occurrence of complications [3, 4]. One study conducted in an adult ICU showed no impact on the modulation of systemic inflammation [5]. Other data from literature mainly reports total parenteral nutrition-associated pulmonary complications [6, 7].

Mechanisms of particle-induced organ dysfunction have generally been investigated in animals [8]. Particles may induce occlusive micro-thrombi, activation of platelets and neutrophil granulocytes, and the formation of granulomas. It has been reported in experimental studies that particles can induce deleterious clinical effects such as thrombogenesis [9] and microcirculation impairment [10]. In critically ill patients, drug incompatibilities frequently occur because of the number of drugs to be administered through an inadequate number of infusion lines [11]. These are among the main causes of particulate contamination. A particulate load induces clinical consequences which have been well described in a previous systematic review [1], indicating how the lungs are particularly affected. The authors demonstrated that around one third of injected particles are trapped in the lungs or tend to locate there primarily [12]. These microparticles caused by drug incompatibility may lead to micro-emboli and granuloma in the pulmonary vessels, particularly during total parenteral nutrition.

The difference in clinical outcomes between children and adults when infused with or without filter poses the problem of exposure level. However, little data is available to quantify particle exposure during simultaneous IV-drug infusion. A dynamic particle count test was used recently to evaluate in vitro the amount of particulate matter potentially administered to patients in a neonatal ICU [13] and a paediatric haematology unit [14]. This method appeared promising and enabled us to assess particulate exposure during continuous drug infusion in critically ill adult patients.

The objective of this study was therefore to evaluate the particulate matter potentially administered to critically ill patients by reproducing in vitro the most common intravenous system and drug combinations used in ICUs for patients suffering from either septic shock or acute respiratory distress syndrome (ARDS).

## Methods

### Experiments, devices and drugs

Observation was made of the most common infusion lines and drugs used on 20 patients admitted for septic shock or ARDS to an adult ICU at Lille University Hospital. A standard infusion protocol quantified the number of particles generated during a simulated 6-h infusion period. Each protocol was repeated six times.

**Fig. 1:**
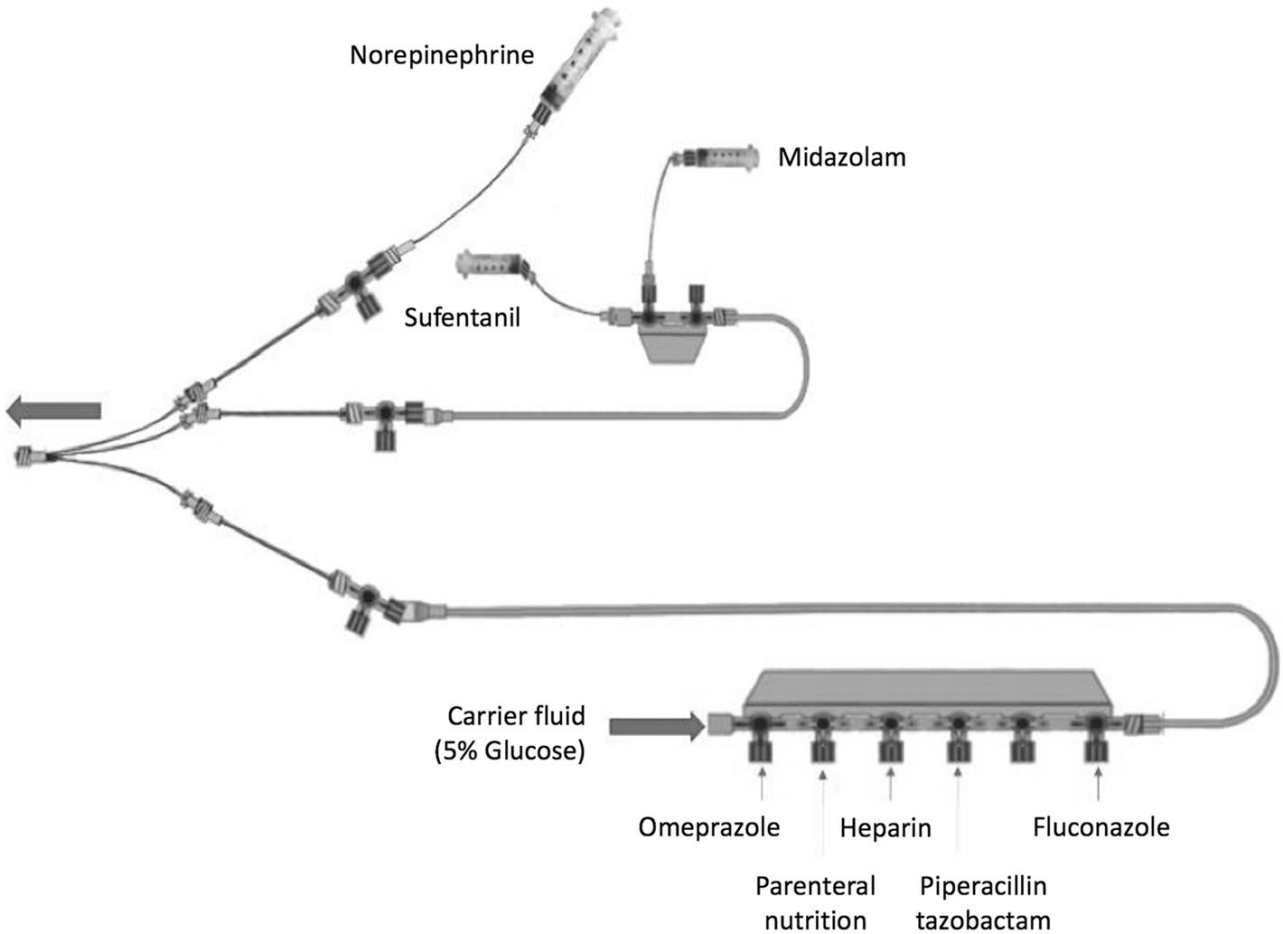
Representation of the IV protocol commonly used in our adult ICU to manage septic shock or ARDS

The three-lumen catheter was replaced by a three-lumen extension set (ref. PY3101KR, Cair LGL, Lissieu, France) so as to connect the infusion line to the particle counter (Fig. 1).

Each lumen of this three-lumen extension set was connected to an extension set with a stopcock (length 25 cm, diameter 2.5 mm, ref. PSS3302MDE, Cair LGL, Lissieu France). At the proximal access, the extension set was connected via the stopcock to another extension set (length 150 cm, diameter 1 mm, ref. PB3115, Cair LGL, Lissieu, France) connected to the norepinephrine syringe. At the medial access, the extension set was connected via the stopcock to another extension set and a two-port manifold (length 200 cm, diameter 2.5 mm, ref. RPB2320, Cair LGL, Lissieu, France) connected to the midazolam and sufentanil infusion line (ref. PSS3302MDE, Cair LGL, Lissieu, France). At the distal access, the extension set was connected via the stopcock to an extension set (length 200 cm, diameter 2.5 mm) and a six-port manifold (ref. RMB6320, Cair LGL, Lissieu, France). Other drugs, 5% glucose and binary parenteral nutrition (PN), were connected to this manifold via pump infusion lines (ref. Z072810F, Fresenius-Kabi, Sèvres, France). Drugs were infused at a fixed concentration through pumps or syringe pumps (DPS module Orchestra®, Fresenius-Kabi, Sèvres, France) following the clinical practices observed (Table 1).

**Table 1:** Drugs Studied

Drug	Final concentrations and/or volume (solvent for dilution)	Infusion flow rate	Time of infusion
Norepinephrine *Noradrénaline Mylan 2 mg/mL* *batch H2028*	24 mg/48 mL (5% glucose)	4 mL/h	Over 6 h (start in 1st position)
Midazolam *Midazolam Mylan 5 mg/mL* *batch F3108*	100 mg/50 mL (0.9% sodium chloride solution)	10 mL/h	Over 6 h (start in 2nd position)
Sufentanil *Sufentanil Mylan 5mcg/mL* *batch H3093*	250 μg/50 mL (0.9% sodium chloride solution)	4 mL/h	Over 6 h (start in 3rd position)
Heparin *Héparine sodique Panpharma 5000UI/mL* *batch 70577*	5000ui/50 mL (0.9% sodium chloride solution)	4 mL/h	Over 6 h (start in 4th position)
5% Glucose *Glucose Baxter 5%* *batches 18A08G64 and 17L19T4A*	500 mL	20.8 mL/h	Over 6 h (start in 5th position)
Binary PN *Olimel N7 Baxter* *batch 17CON950*	1200 mL for amino acid and glucose solutions	62.5 mL/h	Over 6 h (start in 6th position)
Omeprazole *Oméprazole Mylan 40 mg* *batch T523*	40 mg/50 mL (5% glucose)	100 mL/h	T0 + 3 h (30 min)
Piperacillin/Tazobactam *Piperacilline tazobactam Mylan 4 g/0.5 g* *batch 5M2832FR*	4 g/100 mL (5% glucose)	200 mL/h	T0 + 3 h30 (30 min)
Fluconazole *Fluconazole Kabi 2 mg/mL* *batch 5LA229F5*	400 mg/200 mL	400 mL/h	T0 + 4 h (30 min)

### Instrumentation and particle analysis

The egress of the IV line was connected to a Qicpic® dynamic image analysis device (Sympatec GmbH Inc., Clausthal-Zellerfeld, Germany) linked to a Lixell module (Sympatec GmbH). The Qicpic® particle analyser with Windows 5.0 software also determines particle sizes of between 1 μm and 30 mm and provides dynamic imaging analysis [13].

The pH of each different drug solution and the pH of the solution at the egress of the Qicpic® analyser were measured three times with a pH meter (SB70P Symphony®, VWR International, Singapore).

### Data presentation

Particle size characteristics were expressed in Feret’s diameter and length of fibre (LEFI), as described in the USP and EP. Feret’s diameter is the distance between imaginary parallel lines tangent to a randomly oriented particle and perpendicular to the ocular scale. LEFI is defined as the longest direct path from one end to the other within the particle contour and is adapted to non-spherical particles. Particle size distribution (PSD) is described as mass-weighted volume distribution. Shape parameters (sphericity) were used to differentiate air bubbles from particles. Data is expressed in medians (interquartile range), if not otherwise specified.

## Results

The pH of the drug solutions at the final concentration ranged from 3.16 for midazolam at 100 mg/50 mL in a 0.9% sodium chloride solution to 9.63 for omeprazole at 40 mg/50 mL in 5% glucose (Table 2).

**Table 2:** Mean pH of the drug solutions at their final concentration

Drug	Final concentrations and/or volume (solvent for dilution)	Mean pH at the final concentration (standard deviation)
Norepinephrine	24 mg/48 mL (5% glucose)	3.42 (0.05)
Midazolam	100 mg/50 mL (0.9% sodium chloride solution)	3.16 (0.09)
Sufentanil	250 μg/50 mL (0.9% sodium chloride solution)	5.44 (0.13)
Heparin	5000ui/50 mL (0.9% sodium chloride solution)	5.90 (0.09)
5% glucose	500 mL	4.16 (0.17)
Binary parenteral nutrition	1200 mL	5.95 (0.06)
Piperacillin/tazobactam	4 g/100 mL (5% glucose)	5.49 (0.10)
Fluconazole	400 mg/200 mL	5.56 (0.22)
Omeprazole	40 mg/50 mL (5% glucose)	9.63 (0.24)

For the six experimental procedures, the overall number of particles over the 6-h infusion period was 8256 (4725). For the number of particles ≥ 10 and 25 μm, the collected values were 281 (225) and 19 (21) respectively.

**Fig. 2:**
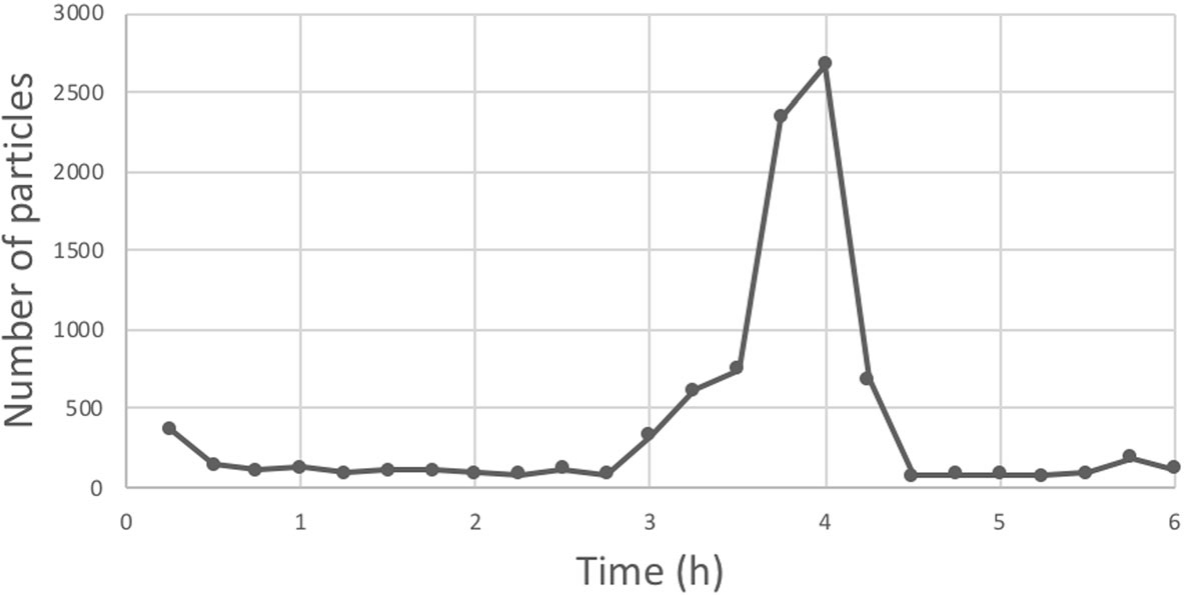
Trend in particulate contamination over time

Our results showed that discontinuous administrations of drugs (omeprazole, piperacillin/tazobactam, and fluconazole) led to disturbances in particulate contamination, resulting in increased particle release between T0 + 3 h and T0 + 4 h30 with 76% of the total number of particles recorded (Fig. 2).

**Fig. 3:**
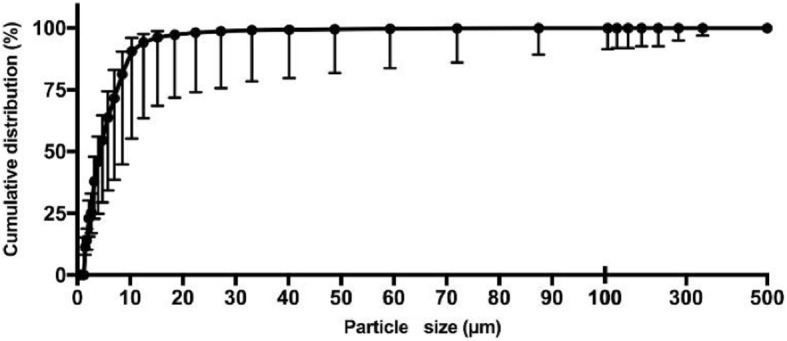
Cumulative distribution of particle size. Data is presented as means with standard deviation

Particle size ranged from 1 to 341 μm with the majority between 1 and 25 μm (> 99% of the total number of particles) (Fig. 3).

## Discussion

Our study is the first to quantify the particulate matter potentially administered to critically ill adult patients during a standard infusion protocol for the treatment of septic shock or acute respiratory distress syndrome. Previous studies have explored particulate contamination during leukaemia treatment in children [14] and NICU multidrug IV therapies [13].

In our case, the particulate load was evaluated over a 6-h infusion period which can be extrapolated to a 24-h period and our results show lower particulate contamination than observed in previous studies [13, 14]. Perez et al. investigated the number of particles received by preterm infants over 24 h during multidrug IV therapies [13]. Two different protocols commonly used in an NICU were reproduced in vitro and analysed with the same equipment as that described in our study. Their results were twice as high as ours.

The main hypothesis accounting for this difference is the reduced contact time between drugs infused simultaneously due to higher drug flow rates. Indeed, Perez et al. indicated that by reducing contact time, a multi-lumen infusion set reduced particulate contamination. In their in vitro study, the authors reproduced the parenteral multidrug infusion used in a paediatric haematology unit [14]. With a multi-lumen infusion set, particulate contamination was significantly reduced by more than two-thirds compared to the standard infusion set. The three-lumen catheter simulated in our experiments by a three-lumen extension set may have a similar effect on drug incompatibility to that of the multi-lumen infusion set. However, the total flow rate was definitely higher in our experiments than in previous experiments in children. In their study, Perez et al. also explored the impact of the concentration of certain drugs and their flow rates. Indeed, the lowest concentration tested with the highest flow rate made it possible to infuse vancomycin and piperacillin-tazobactam without any visible precipitate with either IV administration set [14].

Another result is the impact of discontinuous infusions on particle contamination. Thirty-minute infusions of omeprazole, piperacillin-tazobactam and fluconazole led to disturbances in particulate contamination resulting in increased particle release. Discontinuous infusions lead to changes in total flow rate inducing a transient increase in particle flow and changes in the pH of the solution inducing drug precipitation. This phenomenon was also described by Perez et al. These findings are in accordance with the results of Mehrkens et al. who showed that bolus injections mainly increase the particulate contamination of the fluids administered [15]. As a result, clinicians should be aware of the administering conditions of discontinuous IV drugs and of the effects of changes in carrier or drug on drug delivery during multi-infusion therapy. In our work, the three discontinuous infusions were administered successively through an infusion line connected to a three-lumen extension set that was receiving simultaneously drugs infused from proximal and medial accesses. This configuration reduced the contact time between drugs, but it was not sufficient to prevent the transient increase in particle release. Flushing the infusion line with a neutral fluid between the administration of two incompatible drugs may be a supplemental approach to consider [16].

Our work does not answer the question of which contamination levels can be considered acceptable. It does however provide contamination level benchmarks with regard to clinical studies already published. In a recent systematic review, we described the clinical consequences of drug incompatibilities [1] through case reports and randomised controlled trials. Pulmonary toxicity was reported particularly during prolonged total parenteral nutrition [17] and related to the occurrence of a micro-emboli of crystal precipitates obstructing pulmonary vessels and generating granulomatous pulmonary arteritis and granulomatous interstitial pneumonitis [18]. In critically ill children, in-line filtration reduced the incidence of organ dysfunctions (respiratory, renal and haematological dysfunctions) and sepsis and so demonstrated indirectly the negative effects of particles [2]. Our results may explain the differences in those observed in randomised controlled trials that assess the effect of in-line filtration on the development of sepsis and organ failure. In-line filtration seems an effective strategy for preventing sepsis in children but contrasts with the negative results in adults shown by Gradwolh-Matis et al. [5]. The discrepancies in results between clinical trials in a paediatric ICU and an adult ICU suggest that particulate contamination is more important in critically ill paediatric patients than in critically ill adult patients and could confirm the advantage of in-line filtration in the latter. However, clinicians must take into account that all drugs cannot be filtered and that the use of filters may sometimes lead to drug retention [19].

Orbegozo et al. observed that microvascular reactivity is quickly affected in patients with ARDS, and this impairment is directly related to the severity of the disease. We can therefore easily understand that particles may aggravate sepsis and ARDS; that is the reason why we selected, in our clinical observational study, septic and ARDS patients whose microcirculation is more sensitive to particles [20]. However, the contamination rates found in our study are not completely in line with this hypothesis.

There are certain limitations to this study, the first being the detection range. The particulate counter analysis has a detection limit of 1 μm, which underestimates the number of particles really infused to patients. The second is the standardising of study conditions in this proof-of-concept study. Experiments were performed in static conditions, with precise positioning of the devices and with no disturbance along the infusion line; our assessment was limited to a specific drug combination; parenteral nutritional support was binary and propofol was not studied because our dynamic image analysis instrument was inadapted and not validated for use with lipids. Results may vary with other drug combinations. A similar study including lipid infusion will be conducted to clarify previously described observations of aggregation formation during total PN infusion [6, 7]. The third limitation is that the origin of particles was not explored. There are different causes of particulate contamination of IV fluids. It can occur during the manufacture or preparation of drug solutions or result from drug incompatibility [21]. It may be relevant to assess the extent to which particles originate from the reconstitution of injectable drugs. The fourth is the absence of any assessment of microbiological quality, which would have been useful to further characterise the integrity of the administration approach.

## Conclusions

This work indicates the amount of particulate matter potentially administered to critically ill adult patients. Particulate contamination appears lower than previous measurements taken during multidrug IV therapies in children.

It would be interesting to continue this quantification process and systematically measure particulate contamination during RCTs. The effect of in-line filtration on the development of sepsis should be assessed so as to estimate patients’ potential exposure to contamination and be able to determine exposure thresholds.

## Abbreviations

ARDS: Acute respiratory distress syndrome
ICU: Intensive care unit
IV: Intravenous
LEFI: Length of fibre
NICU: Neonatal intensive care unit
PN: Parenteral nutrition
PSD: Particle size distribution
RCT: Randomised controlled trial
